# Static Softening Behavior and Modified Kinetics of Al 2219 Alloy Based on a Double-Pass Hot Compression Test

**DOI:** 10.3390/ma13173862

**Published:** 2020-09-01

**Authors:** Lei Liu, Yunxin Wu, Hai Gong, Abdulrahaman Shuaibu Ahmad, Fang Dong, Huamin Yu

**Affiliations:** 1 Light Alloy Research Institute, Central South University, Changsha 410083, China; gonghai@csu.edu.cn (H.G.); dongfang@csu.edu.cn (F.D.); guoying623@csu.edu.cn (H.Y.); 2 State Key Laboratory of High-Performance Complex Manufacturing, Central South University, Changsha 410083, China; abddawn@csu.edu.cn; 3 Department of Mechanical Engineering, Kano University of Science and Technology, Wudil 713101, Nigeria

**Keywords:** Al 2219 alloy, static softening mechanism, step ratio, sub-grain boundary, static recrystallization kinetics

## Abstract

In this paper, the static softening mechanism of a 2219 aluminum alloy was studied based on a double-pass isothermal compression test. For the experiment, different temperatures (623 K, 723 K, and 773 K), strain rates (0.1/s, 1/s, and 10/s), deformation ratios (20%, 30%, and 40%), and insulation periods (5 s, 30 s, and 60 s) were used. Based on the double-pass flow stress curves obtained from the experiment, the step rate expressed by the equivalent dynamic recrystallization fraction is dependent on the deformation parameters, which increases with the increase in strain rate and insulation time, while it decreases with the increase in temperature and strain. Based on the microstructure observed using electron backscattered diffraction (EBSD), the static softening mechanism of the Al 2219 alloy is mainly static recovery and incomplete static recrystallization. A new expression for the static recrystallization fraction is proposed using the reduction rate of the sub-grain boundary. The dependent rule on the deformation parameters is consistent with the step rate, but it is of physical significance. In addition, the modified static recrystallization kinetics established by the new SRX fraction method was proven to have a good modeling and prediction performance under given deformation conditions.

## 1. Introduction

The transition ring of the rocket tank is the key component that connects the fuel tanks and bearing stress, and its material selection and forming process has always been a serious challenge [1,2,3]. Currently, the Al 2219 alloy has been widely used as the base material of the aforementioned part, because of its high strength, good weldability, and stable mechanical properties at −250–300 °C [4,5,6,7,8]. The forming of the transition ring mainly goes through technological processes such as casting, forging, and ring rolling, in which multi-pass characteristics are obviously observed, and its good performance is often improved through thermo-mechanical treatment (TMT) [9,10,11]. The softening phenomenon at the deformation intervals is closely related to the deformation process, and it is affected by many complex factors, which will eventually determine the microstructures and the specific performance [12]. Therefore, when enhancing the TMT process and material performance, it is of great significance to focus on the softening mechanism of the material, as well as its corresponding static recrystallization kinetic model.

Presently, the research on static softening behavior is mainly based on double-pass thermal compression experiments [13,14,15], which have been widely conducted in various metals and alloys, such as Al-Zn-Mg-Cu alloys with various Zr additions [16], alloyed steel [17], titanium added ultra-low carbon (ULC-Ti) steel [18], 7150 aluminum alloy [19], Al–6Mg alloy [20], and vanadium micro-alloyed high manganese austenitic steels [21]. The reports on static softening mainly focus on the softening mechanism and kinetics modeling. For the mechanism part, Z. Jin et al. [22] found that meta-dynamic recrystallization (MDRX) and static recrystallization (SRX) occurred during the deformation interval of the AZ31 alloy. M. Zhao et al. [23] pointed out that the softening mechanism of 300M steel is mainly static recovery (SRV) and SRX as a result of strain-induced boundary migration (SIBM) through in-situ observation. X. Xu et al. [24] explored the effect of Al on the recovery of low-density ferritic steel containing 4 mass% aluminum, and explained the phenomenon of the suppressed recovery and correspondingly the promoted recrystallization. G. Bo et al. [25] studied the static softening behavior of a Al–Cu–Mg–Zr alloy under different pre-precipitated microstructures, and interpreted the double plateaus in the static softening curve as static recovery, static precipitation, and coarsening, as well as the complete depletion of stored energy during the preserving process. For the modeling, F. Jiang et al. [26] established an empirical static recrystallization model of a 7150 aluminum alloy based on the Johnson–Mehl–Avrami–Kolmogorov (JMAK) equation. J. Tang et al. [27] constructed a simplified static softening model of a Al-Zn-Mg-Cu alloy coupled with SRV and SRX. A. Yanagida et al. [28] proposed a formulation method for a softening fraction (SF), and established static recrystallization kinetics of plain carbon steel by reverse analysis. W. Shen et al. [29] established modified Avrami static recrystallization kinetics of Nb-V micro-alloyed steel, which regarded the material parameter (n) as the deformation parameter dependent. Although numerous kinetics of static softening have been established, most of them are derived indirectly from stress–strain curves, and few are derived from the statistics result of the microstructures, which may lead to some deviation on the accuracy of the models.

In this paper, the static softening mechanism of the 2219 aluminum alloy based on the double-pass thermal compression test and the factors influencing it were studied. Finally, the revised kinetics for the static recrystallization was proposed and established according to the specific physical mechanisms and micrographs.

## 2. Materials and Methods

**Figure 1:**
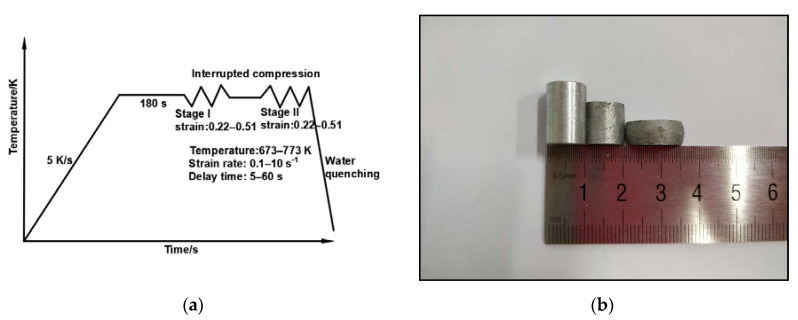
(**a**) Double-pass hot compression test; (**b**) the samples before and after the experiment.

An Al 2219 alloy with the composition of Al-(5.8–6.8)Cu-(0.2–0.4)Mn-0.2Si-(0.1–0.25)Zr-0.3Fe-0.02Mg-0.1Zn-(0.05–0.15)V-(0.02–0.1)Ti (weight pct.) was utilized in this test; the base material was taken from a part that was processed via post hot forging and solid solution treatment (SST) for 4 h. The samples were machined into cylinders with a diameter of 10 mm and a height of 15 mm. The two ends of the samples were coated with boron nitride and mica in order to reduce the friction between the sample and the pressure head. The test was conducted using the Gleeble-3500 thermal-mechanical testing system, based on the process curve shown in Figure 1a. The experimental procedure was as follows: The samples were heated at the set temperatures (623 K, 723 K, and 773 K) at a heating rate of 5 K/s. As the peak temperature was attained, the samples were held for 180 s to obtain a uniformly distributed temperature. Then the first-pass compression test was carried out using (20%, 30%, and 40%) deformation ratios and strain rates of (0.1/s, 1/s, and 10/s) respectively, followed by isothermal heat preservation for a duration of (5 s, 30 s, and 60 s). Then, the metallographic samples for the microstructure examination were cut, while the second-pass compression test was performed on the remaining specimens, in which the strain rate used in the first-pass was maintained, while the deformation ratios were changed to 40%, 30%, and 20%, so that the total deformation ratios of the three samples would be the same. Water quenching was performed immediately after the second-pass thermal compression test in order to preserve the deformed microstructure for the subsequent metallographic investigations. The samples before and after the test are presented in Figure 1b. After compression, the samples were cut parallel to the compression direction, and the obtained planes were observed using the electron backscattered diffraction (EBSD) and scanning electron microscope (SEM) techniques. The samples for SEM were ground and mechanically polished, while the EBSD samples were ground and electropolished (methanol nitrate solution, voltage 20 V, and time 50 s). The data obtained from the EBSD test were processed using Channal5 software, where a high angle grain boundary (HAGB; misorientation > 15°) was represented by a thick black solid line, while low angle grain boundary (LAGB; 2° < misorientation < 15°) was represented by a fine white solid line.

**Figure 2:**
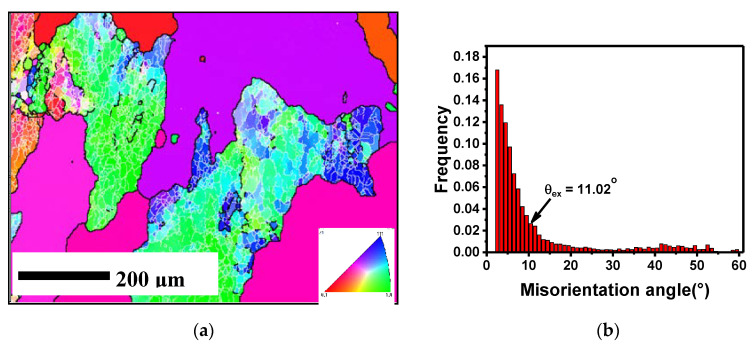

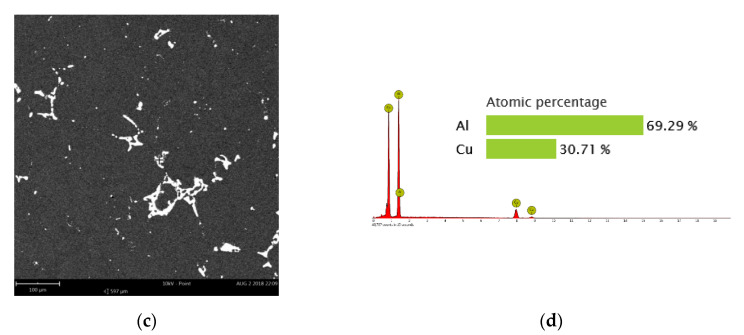
Initial state of the deformed samples: (**a**) optical microstructure (OM) and grain boundary (GB) figure; (**b**) misorientation angle frequency; (**c**) SEM micrograph; (**d**) energy disperse spectroscopy (EDS) analysis.

The initial microstructure of the Al 2219 alloy used before the compression test is shown in Figure 2. As presented in Figure 2a, the grain size after SST was relatively large (>200 μm). Furthrmore, some grains were densely covered with a large number of sub-structures, while some grains were completely devoid of sub-structures, which indicates that SST could only restore part of the sub-structures, but could not completely eliminate it. In addition, small recrystallized grains were still left in the local area. As shown in Figure 2b, the LAGB had a proportion of 81.7%, while the average misorientation of the initial state was 11.02°. It can be seen from Figure 2c that some precipitates clustered together and existed as chains in the aluminum matrix. From Figure 2d, the second phase was determined to be CuAl_2_ (θ) particles using energy disperse spectroscopy (EDS) analysis.

The initial microstructure of the Al 2219 alloy used before the compression test is shown in Figure 2. As presented in Figure 2a, the grain size after SST was relatively large (>200 μm). Furthermore, some grains were densely covered with a large number of sub-structures, while some grains were completely devoid of sub-structures, which indicates that SST could only restore part of the sub-structures, but could not completely eliminate it. Also, small recrystallized grains were still left in the local area. As shown in Figure 2b, LAGB had a proportion of 81.7%, while the average misorientation of the initial state was 11.02°. It can be seen from Figure 2c that some precipitates clustered together and existed as chains in the aluminum matrix. From Figure 2d, the second phase was determined to be CuAl_2_ (θ) particles using energy disperse spectroscopy (EDS) analysis.

## 3. Results and Discussion

### 3.1. Static Softening Mechanism

#### 3.1.1. True Stress–Strain Curve

**Figure 3:**
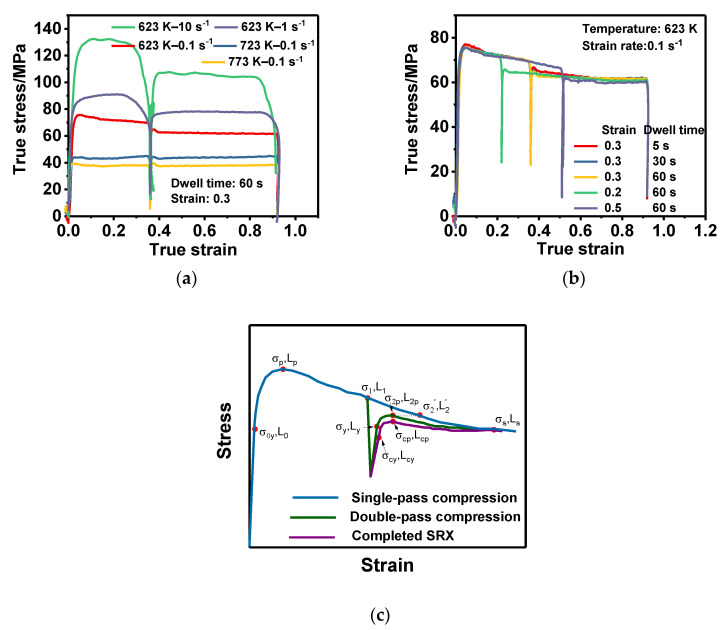
True stress–strain curves under the condition of (**a**) different temperatures and strain rates at the same dwell time and strain; (**b**) different dwell times and strains at the same temperature and strain rate; (**c**) single-pass, double-pass, and completed static recrystallization (SRX) compression.

The true stress–strain curves obtained from the experiment are shown in Figure 3. It can be seen that the work-hardening stage was very short (within 0.05 strain), because the initial state still contained a large number of sub-structures, so the critical point of dynamic recovery (DRV) could be reached very quickly [30]. Moreover, the flow curve showed a trend of gradually decreasing in each pass. According to the previous study of the author [31], this was because of the occurrence of the continuous dynamic recrystallization (CDRX) of the Al 2219 alloy, where the LAGBs gradually accumulated into the HAGBs, and the average dislocation density gradually decreased [32]. Furthermore, the step phenomenon was obvious between the two compression passes. The amount of step deviation was dependent on the deformation parameters (i.e., temperature, strain rate, strain, insulation time, etc.), which indicates that the static softening occurred to different degrees during the intervals [33]. In this paper, the relationship between the flow curve and microstructure was applied to explore the static softening mechanism and quantitative description of the step phenomena. According to the previous study by the author [34], the flow stress was proportional to the (sub)grain boundary content per unit area of the material. Therefore, the decrease in flow stress indicated that the (sub)grain boundary content per unit area decreased according to the flow curve, as shown in Figure 3c, while HAGBs gradually increased at this time by the CDRX mechanism. Hence, it can be concluded that LAGBs gradually decreases in this process. As shown in Figure 3c, the peak stress of the second pass, which was approximately equivalent to the corresponding point of single-pass compression, was less than the interrupted stress of the first pass because of the appearance of the step phenomenon. The difference between the breakpoint and equivalent point in the single-pass curve revealed that a large number of LAGBs disappeared, and new HAGBs were generated. In other words, the occurrence of the step phenomenon proved that not only SRV occured, but SRX also appeared during the interval.

**Figure 4:**
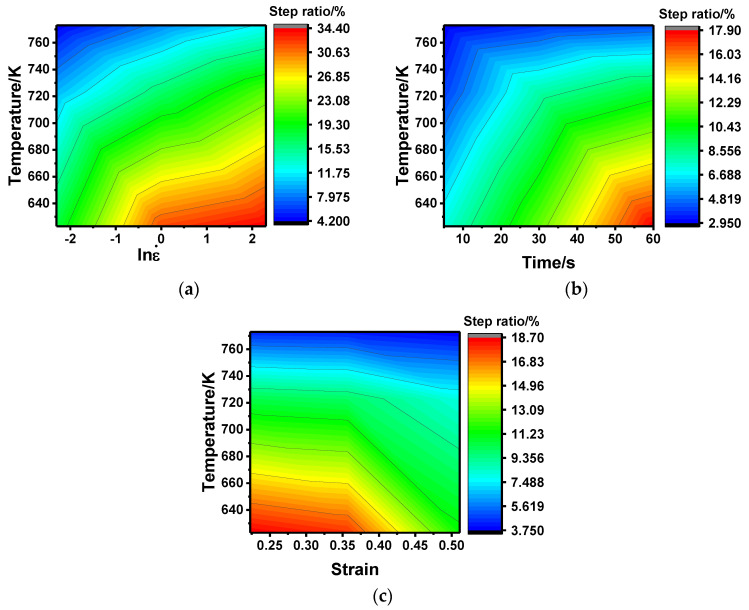
Step ratio under the deformation conditions of different temperatures and (**a**) $\ln\dot{\varepsilon }$, (**b**) time, and (**c**) strain.

Based on the above explanation, the equivalent CDRX fraction was proposed in order to represent the step ratio quantitatively, and the expression is shown as follows.

(1) $$X_{C_{\mathrm{eq}}}\;=\;\frac{\sigma_{1}-\sigma_{2p}}{\sigma_{p}-\sigma_{s}},$$

where σ_1_ is the breakpoint stress (MPa), σ_2p_ is the second-pass peak stress (MPa), σ_p_ is the first-pass peak stress (MPa), and $\sigma_{s}$ is the final steady-state stress (MPa). The step ratio under different deformation conditions can be obtained by Equation (1), and the results are shown in Figure 4. In essence, the step ratio is the proportion of HAGBs generated during the interval when the whole HAGBs are generated for a complete CDRX process of a single-pass compression, which can reflect the static softening rate to some extent. Therefore, it can be seen from Figure 3 that all the step ratios are relatively small, and the maximum value is less than 35%, which indicates that the static softening effect during the interval is not very significant. In addition, it is also obvious that the step ratio increased with the increase in strain rate and holding time, while it decreased with the increase in strain and temperature. This is because a large number of sub-grain boundaries were generated with an increase in strain rate. The stored energy in the sub-grain boundary could drive the movement of the sub-grain boundary during the thermal insulation period, resulting in the gradual accumulation of LAGBs’ misorientation to produce HAGBs. In addition, prolonged thermal insulation time could promote this transformation process, and the rise in temperature could improve the kinetic energy of the atoms and speed up the dislocation movement, while the high temperature made the content of the grain boundary (GB) reserve less, leading to the abate of the GB moving force. Evidently, the two effects were to the contrary. It can be inferred that the consequences of the reduced sub-grain boundary were greater than the activation ascension, as a result of the increasing temperature. Therefore, the static softening effect was reduced with the increase in temperature. Finally, the LAGBs contents gradually decreased with the increase in strain, leading to the weakening of the driving force during the insulation process and the static softening rate.

#### 3.1.2. Microstructure Evolution

**Figure 5:**
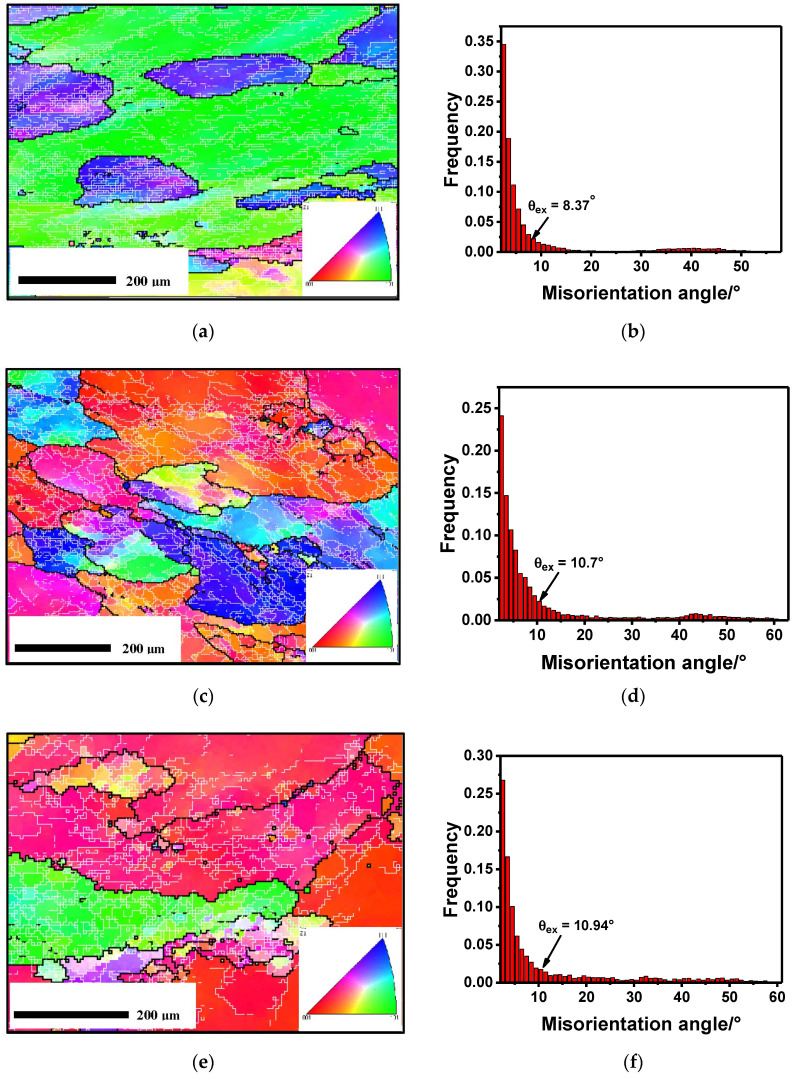
OM and GB figures under the conditions of: (**a**) 623K–0.1/s–60s–0.36; (**c**) 723K–0.1/s–60s–0.51; (**e**) 773K–0.1/s–60s–0.36. Misorientation angle frequency under the conditions of: (**b**) 623K–0.1/s–60s–0.36; (**d**) 723K–0.1/s–60s–0.51; (**f**) 773K–0.1/s–60s–0.36.

The microstructures obtained for different deformation conditions after the thermal insulation stage are shown in Figure 5. It can be seen that the deformed grains were still covered with a large number of sub-grain boundaries, and many large sub-grains were observed, indicating that SRV caused by sub-grain boundary migration occured in the intermediate softening stage. Moreover, some scattered non-closed HAGBs were observed inside the grains, which proves that SRX also occured by the transformation of LAGBs due to the accumulation of misorientation. Apparently, the SRX ratio was very small and was also affected by the deformation parameters. In addition, the statistics of the HAGB content (L_HAGB_ and η_HAGB_), and the mean misorientation angle (θ*_ex_*) under different conditions are shown in Table 1. It can be seen that the HAGB content increased when compared with the initial state, indicating that recrystallization occured, but it is not clear whether it was generated in a dynamic process or static state. Also, the HAGB and average misorientation angle under the different deformation conditions shown in Table 1 could not be directly compared and no conclusion could be be derived. This is because they experienced different complex evolutions under different deformation conditions, which generated different amounts of sub-grains and misorientation, meaning they had different initial states before the isothermal insulation stage. To establish the relationship between the static softening and deformation parameters, a new independent expression method was proposed, which is described in detail below.

**Figure 6:**
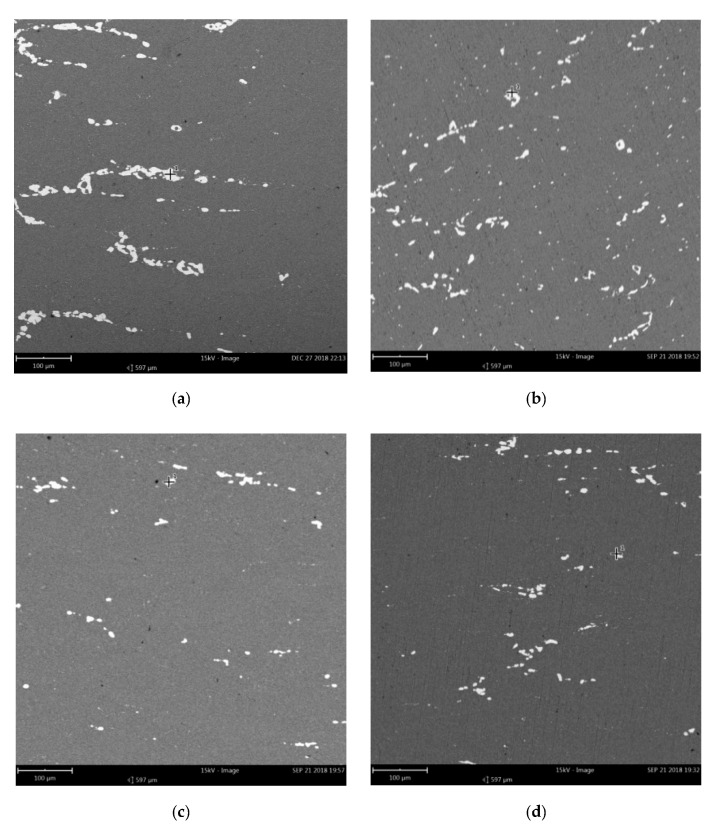

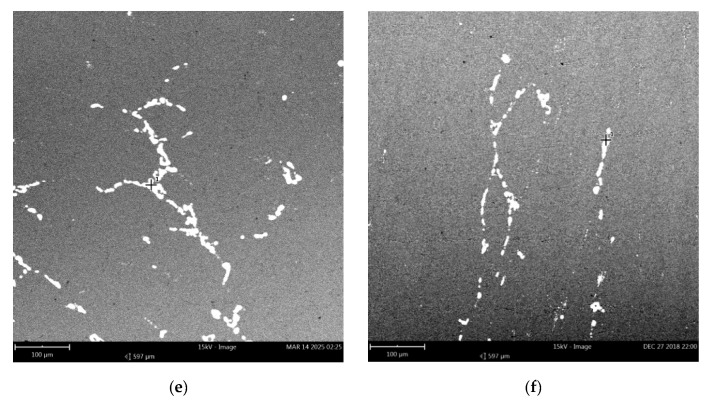
SEM micrographs of the deformed samples under the conditions of: (**a**) 623K–0.1/s–60s–0.36; (**b**) 623K–0.1/s–60s–0.9; (**c**) 723K–0.1/s–60s–0.9; (**d**) 723K–0.1/s–5s–0.9; (**e**) 723K–1/s–60s–0.36; (**f**) 723K–10/s–60s–0.36.

Figure 6 shows the SEM micrographs of the samples under different deformation conditions. It can be seen that the deformation parameters have great influence on the precipitation and evolution of the second phase particles. Figure 6a,b shows the SEM results of the first pass and the second pass with the same deformation parameters, respectively. It can be seen that the total precipitates of the second phase varied slightly with the increase in strain, but the aggregate chain particles were obviously fragmented, indicating that the continuous deformation had a positive effect on the coarse CuAl_2_ particles fragment. Figure 6b,c shows the evolution of the precipitate particles at different temperatures. It is obvious that the precipitation particles decreased with the increase in temperature. This is because the solubility of copper atoms in the aluminum matrix increased with the increase in temperature, and many copper atoms redissolved into the aluminum matrix, resulting in a decrease of precipitation particles. Figure 6c,d shows the precipitation phase at different insulation times. It can be seen that the overall change was not obvious, indicating that the static process had little influence on the precipitation of CuAl_2_ particles when the other conditions remained unchanged. Figure 6e,f shows the morphologies of the precipitated particles at different strain rates. It can be seen that the amount of precipitated particles was similar, and the size of the CuAl_2_ particles decreased slightly at a high strain rate, indicating that the high strain rate was conducive to the fragmentation of precipitated particles.

From a microscopic perspective, the dislocation density and precipitated particles could be applied to qualitatively reveal the softening mechanism of SRX and its dependence on the deformation parameters. At a relatively high temperature, the dislocation density was low, which made the migration drive force of subgrain boundary insufficient, and the coarse precipitates were less, which reduced the nucleation of the particle stimulate nucleation (PSN). Therefore, the high temperature inhibited the development of the static recrystallization. At a relative high strain rate, many dislocations were generated and the precipitates’ content remained almost constant, so at a high dislocation density zone, the coarse precipitates promoted the migration and growth of the subgrain boundary, thus promoting the development of the static recrystallization. At a relative high strain, the dislocation density decreased slightly because of the dynamic recovery and recrystallization, and the size of the coarse precipitates decreased and the distribution was dispersed. Therefore, the drive force of the sub-grain boundary migration was weakened and the growth process was obstructed by the dispersed precipitates, so the development of static recrystallization was decreased with the increase in strain. In addition, with the increase in insulation period, the precipitates remained constant, and the stored dislocation energy accelerated the migration and growth of the sub-grain boundaries, thus, static recrystallization could be more fully developed. Based on the microscopic mechanism discussed above, a quantitative description of the static recrystallization will be presented below.

### 3.2. New Expression for Static Softening Fraction

According to the actual effect of SRX, its fraction is usually represented by the static softening fraction (FS), which is usually represented by the function of flow stress [35,36,37].

(2) $$\mathrm{FS}=\frac{\sigma_{1}-\sigma_{y}}{\sigma_{1}-\sigma_{\mathrm{cy}}}$$

where σ_1_ is the breakpoint stress (MPa), σ_y_ is the second-pass yield stress (MPa), and σ_cy_ is the yield stress after complete static recrystallization (MPa). σ_cy_ is often approximated to σ_0y_, which is significant only if the initial state is fully recrystallized. For this test and most of the commonly implemented tests, their values are not equal, because the initial state of the samples is not always fully recrystallized. Considering that, this method is no longer applicable nor the microscopic transformation mechanism of SRX. Hence, a quantitative description of SRX fraction based on the reduction rate of the LAGBs in the isothermal insulation stage is proposed and is expressed as follows:

(3) $$X_{S}=\frac{L_{1_{L}}-L_{y_{L}}}{L_{1_{L}}}\times 100\%,$$

where X_s_ is the SRX fraction, L_1L_ is the LAGB content at the breakpoint (μm), and L_yL_ is the LAGB content after the holding time (μm).

#### 3.2.1. The Determination of L_1L_

According to the first-pass thermal compression curve, the LAGB content reduction from the peak point to the breakpoint can be obtained by the CDRX fraction, so the value of L_1L_ can be determined by the following equation.

(4) $$L_{1_{L}}=L_{p_{L}}-X_{C}{*L}_{p_{L}},$$

where X_c_ is the first-pass CDRX fraction, and its value in different hot deformation conditions can be obtained from the author’s previously published article [34], and L_PL_ is the LAGB content at the peak point (μm), and its value can be obtained by a reduction in the total GB and HAGB content at the peak point. Based on the microscopic transformation mechanism, the HAGB content for the peak point is equal to that of the initial state, because the peak point appears before the occurrence of DRX, so L_PL_ can be expressed as follows:

(5) $$L_{p_{L}}=L_{p}-L_{0H},$$

where L_p_ is the total GB content of the peak point (μm), and its value can be obtained from the linear relationship between the GB content and flow stress. $L_{0H}$ is the content of the HAGB in the initial state (μm).

#### 3.2.2. The Determination of L_yL_

After the insulation stage, the LAGB content was obtained by the reduction between the total GB and HAGB content at the yield point of the second pass, and can be expressed as:

(6) $$L_{\mathrm{yL}}=L_{y}-L_{\mathrm{yH}},$$

where L_y_ is the total GB content after the thermal insulation stage (μm), and its value can be obtained from the linear relationship between GB content and the flow stress. L_yH_ is the content of HAGB content after the thermal insulation stage (μm). According to Figure 3c, the HAGB content at this point is approximately equal to the value at the peak point of the second pass, which can be regarded as the corresponding equivalent HAGB content ($L_{2H}^{\prime }$). Similarly, L_yH_ can be determined based on the CDRX fraction, and the expression is as follows.

(7) $$L_{\mathrm{yH}}=L_{2}^{\prime }-\left(L_{1L}-X_{C_{\mathrm{eq}}}\cdot L_{\mathrm{pL}}\right),$$

where $L_{2}^{\prime }$ is the total GB content at the equivalent point (μm), and its value can be obtained from the linear relationship between the GB content and flow stress.

**Figure 7:**
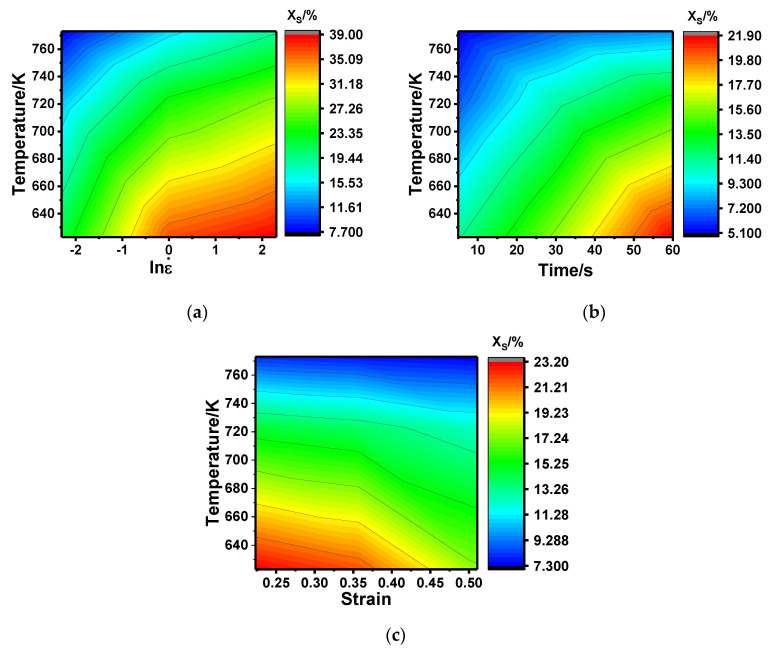
Static recrystallization fraction under the deformation condition of different temperatures and (**a**)$\;\ln\dot{\varepsilon }$, (**b**) time, and (**c**) strain.

Based on the steps above, the SRX fraction under different thermal deformation parameters can be obtained, and the results are shown in Figure 7. As presented in Figure 6, the SRX fraction ranges from 5% to 40% under different deformation conditions, indicating that the SRX marked by the disappearance of the sub-grain boundary occurs. It has, however, was proven to be incomplete static recrystallization because of the small value. Moreover, it can be seen that the SRX fraction increased with the increase in strain rate and insulation time, while it decreased with the increase in temperature and strain, which is consistent with the conclusions of the step ratio above. The numerical differences of the two description methods present two different perspectives to describe the static softening behavior; the former is more inclined to use the increasing rate of the HAGB, while the latter uses the reduction rate of the LAGB. As the ratio of the HAGB generation and the disappearance of LAGB cannot be 1:1 during SRX, it will lead to a deviation between the two methods. As SRX is usually marked by the complete disappearance of the sub-structures, it is more accurate to describe the SRX fraction with the reduction rate of LAGB.

### 3.3. Modified SRX Kinetics

Generally, the SRX kinetics is expressed by the Avrami type equation, as shown below [38,39,40].

(8) $$X_{S}=1-\exp\left[-0.693\cdot {\left(\frac{t}{t_{0.5}}\right)}^{n}\right],$$

where X_s_ is SRX fraction, t is the time (s), t_0.5_ is the time when 50% recrystallization occurs, and n is the material parameter.

#### 3.3.1. Determination of *n*

By taking the logarithm of Equation (8) twice consecutively, the following formula can be obtained.

(9) $$\ln\left[\ln\left(\frac{1}{1-X_{S}}\right)\right]=\ln0.693+\mathrm{nlnt}-{\mathrm{nlnt}}_{0.5},$$

**Figure 8:**
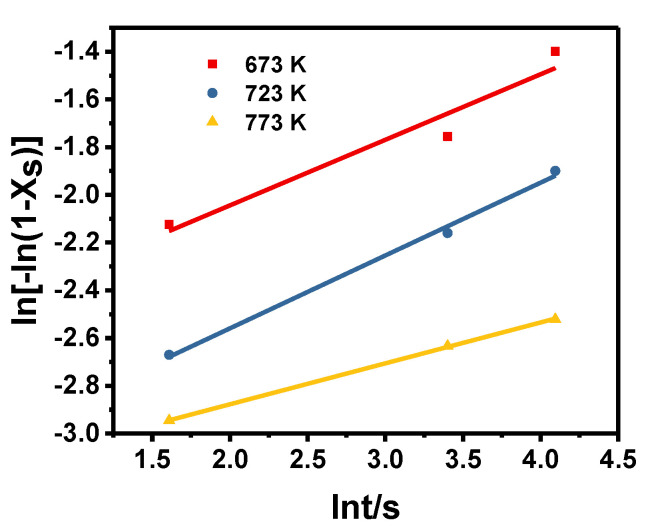
The relationship between ln[−ln(1 − X_S_)] and lnt.

The relationship between $\ln\left[\ln\left(\frac{1}{1-X_{S}}\right)\right]$ and lnt is shown in Figure 8. The value of n can be obtained by the average slope. By linear regression analysis, *n* = 0.251 was obtained.

#### 3.3.2. Determination of t_0.5_

t_0.5_ was determined to be related to the strain rate, deformation temperature, pre-deformation amount, thermal insulation temperature, time, etc., and a mathematical relationship was established with some of them. In this paper, all of these factors were taken into consideration for establishing a comprehensive mathematical relation, as shown below:

(10) $$t_{0.5}=a\cdot {\dot{\varepsilon }}^{b}\cdot \varepsilon^{c}\cdot \exp\left(\frac{{\mathrm{kQ}}_{d}}{{\mathrm{RT}}_{d}}\right)\cdot \exp\left(\frac{{\mathrm{mQ}}_{a}}{{\mathrm{RT}}_{a}}\right),$$

where a, b, c, k, and m are the material parameters; $\dot{\varepsilon }$ is the strain rate; ε is the strain; T_d_ is deformation temperature; T_a_ is annealing temperature; R is the gas constant; and Q_d_ and Q_a_ are the dynamic and static thermal activation energies (kJ/mol), respectively. In this paper, the temperatures (T_d_ and T_a_) were consistent because of the isothermal compression and insulation process. Therefore, Equation (10) can be simplified as follows:

(11) $$t_{0.5}=a\cdot {\dot{\varepsilon }}^{b}\cdot \varepsilon^{c}\cdot \exp\left(\frac{{\mathrm{kQ}}_{d}}{{\mathrm{RT}}_{d}}\right)\cdot \exp\left(\frac{{\mathrm{mQ}}_{a}}{{\mathrm{RT}}_{a}}\right),$$

where Q_m_ is the equivalent thermal activation energy (kJ/mol), and its expression is Q_m_ = kQ_d_ + mQ_a_. By taking the logarithm of Equation (11), the following equation is obtained.

(12) $${\mathrm{lnt}}_{0.5}=\mathrm{lna}+\mathrm{bln}\dot{\varepsilon }+\mathrm{cln}\varepsilon +\frac{Q_{m}}{\mathrm{RT}},$$

**Figure 9:**
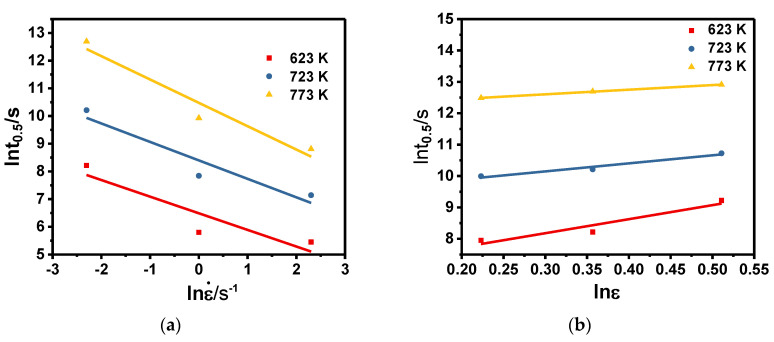

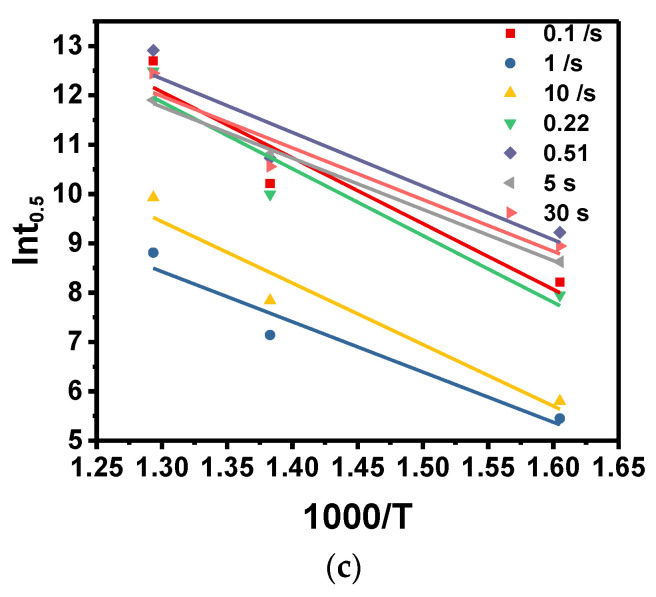
The relationship between lnt_0.5_ and (**a**) $\ln\dot{\varepsilon }$, (**b**) lnε, and (**c**) 1000/T.

The relationship between lnt_0.5_ and lnέ, lnε, and 1/T are shown in Figure 9. Using linear regression, the values of b, c, Q_m_ can be obtained by the average slope, and the results are shown in Table 2. The value of ln a can be obtained by substituting the obtained values into Equation (12).

In summary, the modified SRX kinetics of the Al 2219 alloy were established, and its expression is as follows:

(13) $$\{\begin{array}{c} X_{S}=1-\exp\left[-0.3693\cdot {\left(\frac{t}{t_{0.5}}\right)}^{0.251}\right]\\ t_{0.5}=2.356\times 10^{17}\cdot {\dot{\varepsilon }}^{-0.703}\cdot \varepsilon^{2.835}\cdot \exp\left(\frac{-98.317\times 10^{3}}{\mathrm{RT}}\right) \end{array},$$

**Figure 10:**
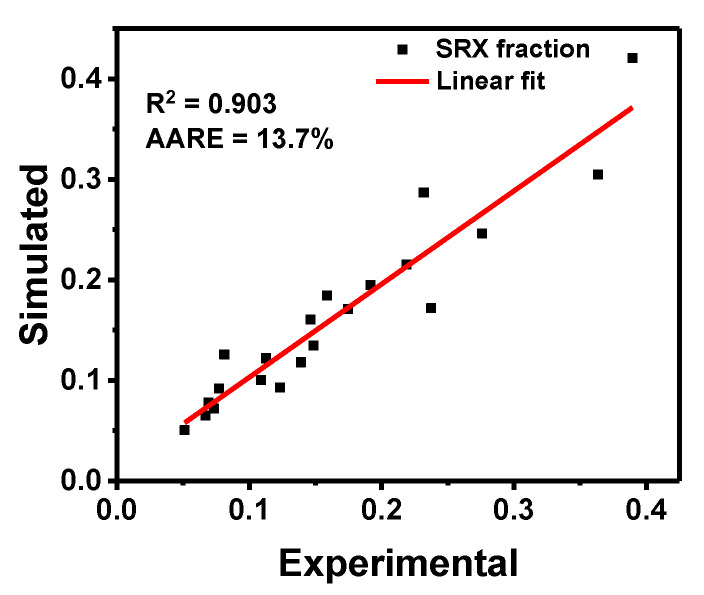
Comparison of the simulated and experimental values of the SRX fraction under different conditions.

To verify the accuracy of the established model, the experimental and simulated values under different deformation parameters are compared in Figure 10. It can be observed that the overall recrystallization fraction was within 40% in the given conditions, and the experimental and simulated data regularity were similar. As shown in Figure 10, the simulated and experimental values were in good agreement with R^2^ = 0.903 and AARE = 13.7%, which indicates that the model was accurate and could be used to predict the SRX fraction under prescribed conditions.

## 4. Conclusions

In this paper, the static softening mechanism of the Al 2219 alloy was studied based on the double-pass thermal compression test, and a modified SRX kinetic model was established by the new expression of the SRX fraction. The specific conclusions are as follows:

- The static softening mechanism of the Al 2219 alloy is mainly SRV and incomplete SRX, which is determined via step phenomenon and microstructure detection;
- The step rate of the first- and second-pass are represented by the equivalent dynamic recrystallization fraction in the form of flow stress, which increases with the increase in strain rate and isothermal insulation time, while it decreases with the increase in temperature and strain;
- A new expression for the SRX fraction is proposed based on the reduction rate of the sub-grain boundary. Compared with the traditional stress method, the new expression method is derived from the micro perspective, and the data obtained from experiment and EBSD observation are more real and effective, which is feasible in theory and practice and is applicable to samples of all processing states. The dependent rule on deformation parameters is consistent with the step rate, and it is of physical significance;
- The SRX kinetic model considering all of the deformation factors established in this paper has good modeling and prediction performance under the given deformation conditions, with a correlation coefficient of 0.903 and a relative error of 13.7%, and the method can be applied to other similar materials.

## Figures and Tables

**Table 1:** Material parameters under different conditions after the thermal insulation stage.

Material Parameters	623K–0.1/s–5s–0.36	623K–0.1/s–60s–0.36	723K–0.1/s–60s–0.22	723K–0.1/s–60s–0.51	773K–0.1/s–60s–0.36	773K–10/s–60s–0.36
L_HAGB_/μm	9.261 × 10^3^	1.529 × 10^4^	1.046 × 10^4^	1.568 × 10^4^	1.152 × 10^4^	1.245 × 10^4^
η_HAGB_	0.127	0.226	0.189	0.275	0.217	0.176
θ_ex_	8.035	8.37	8.74	10.704	10.942	9.171

**Table 2:** The values of the material parameters.

Parameters	b	c	Q_m_	lna
Values	−0.703	2.835	−98.317 × 10^3^	28.567
